# Three cases of endoscopic submucosal dissection for vocal fold carcinoma and precancerous lesions

**DOI:** 10.1055/a-2860-0370

**Published:** 2026-05-22

**Authors:** Qingmiao Zhao, Yueming Zhang, Shuying Guo, Yong Liu, Ziyue Wang, Shun He, Guiqi Wang

**Affiliations:** 1 Department of Endoscopy, National Cancer Center/National Clinical Research Center for Cancer/Cancer Hospital 12501 Chinese Academy of Medical Sciences and Peking Union Medical CollegeBeijingChina; 2 Department of Pathology, National Cancer Center/National Clinical Research Center for Cancer/Cancer Hospital 12501 Chinese Academy of Medical Sciences and Peking Union Medical CollegeBeijingChina

Laryngeal carcinoma accounts for one-third of all head and neck cancers and is associated with high morbidity and mortality, classified into supraglottic, glottic, and subglottic types 1 . Vocal fold leukoplakia is the most common precancerous condition of the vocal fold 2 . Surgery is the primary treatment for vocal fold leukoplakia (with high-grade dysplasia or carcinoma in situ) as well as early-stage vocal fold carcinoma. Transoral laser microsurgery (TLM) remains the standard approach 1 2 . However, TLM is an ablative procedure that does not allow definitive histopathological evaluation, thereby limiting guidance for subsequent follow-up and additional therapy.

For the first time, we report two cases of vocal fold precancerous lesions and one case of vocal fold carcinoma that were safely and successfully treated with endoscopic submucosal dissection (ESD). Here, we present the complete diagnostic and therapeutic process, including intraoperative images and a video, of one representative case of vocal fold leukoplakia.

**Fig. 1:**
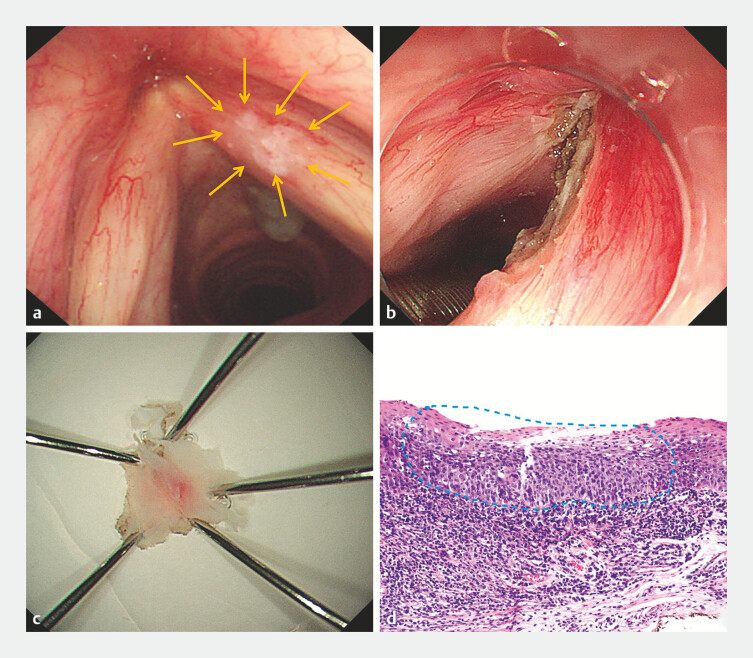
**a** A right vocal fold leukoplakia (a cycle of yellow arrows surrounds the lesion). **b** A defect in the right vocal fold after endoscopic submucosal dissection. **c** The specimen removed en bloc. **d** A histopathological image of the lesion (the blue dashed line outlines the extent of high grade intraepithelial neoplasia).

A 56-year-old man was diagnosed with right vocal fold leukoplakia ( Fig. 1 **a** ) via laryngoscopy and biopsy during follow-up after esophageal ESD. Contrast-enhanced computed tomography revealed no cervical lymph node metastasis. Given the high-risk of the lesion (showing severe dysplasia on biopsy) and the patient’s desire for definitive histopathological assessment, ESD was performed. The lesion was resected en bloc in accordance with standard ESD procedures, including marking, submucosal injection, submucosal dissection, and electrocoagulation for hemostasis ( Fig. 1 **b, c** , Video 1 ). The procedure lasted 30 minutes.

Endoscopic Submucosal Dissection for vocal fold precancerous lesion.Video 1

**Fig. 2:**
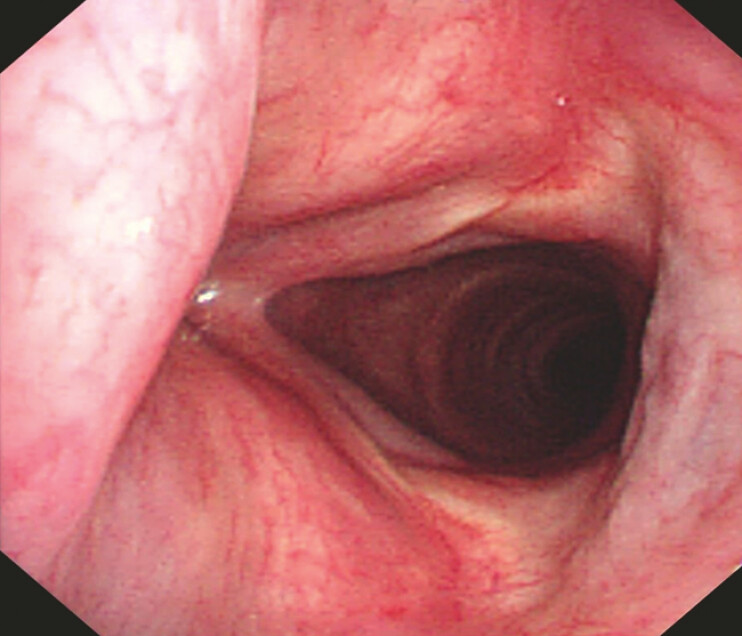
The defect on postoperative month 36.

**Fig. 3:**
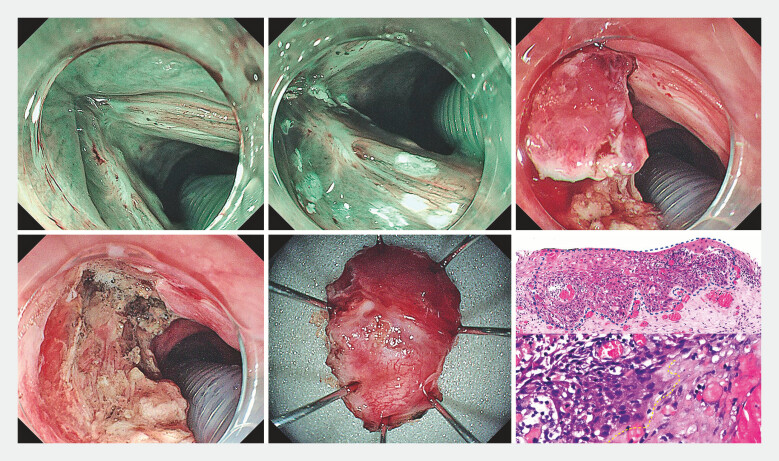
Another case of vocal fold carcinoma treated with ESD. ESD, endoscopic submucosal dissection.

Due to the risk of laryngeal edema and airway compromise after vocal fold intervention, the patient was transferred to the intensive care unit postoperatively. On postoperative day (POD) 1, the endotracheal tube was removed. On POD 2, the patient was transferred to the general ward. On POD 3, enteral nutrition via a nasogastric tube was initiated, and intermittent oral rinses with chlorhexidine or lidocaine gel were used for analgesia. On POD 7, the patient was discharged with the nasogastric tube in place, which was planned for removal 1 week later. No adverse events occurred. Histopathological examination confirmed the complete excision of squamous epithelium with severe dysplasia, with tumor-free basal and lateral margins ( Fig. 1 **d** ). At the 3-year follow-up, the patient reported no complications such as hoarseness, lowered pitch, or diminished voice. Nasopharyngoscopy showed satisfactory healing and no recurrence ( Fig. 2 ). In addition, we provide clinical images from another case of vocal fold carcinoma treated with ESD for reference and discussion ( Fig. 3 ).

To date, no previous reports have described the case of ESD for vocal fold carcinoma or precancerous lesions. In our three cases, the lesion was completely removed without compromising the vocal fold structure or function, while enabling precise histopathological evaluation to guide subsequent management. During ESD, protection of adjacent nerves is critical, and the assistant’s role in exposure is essential given the anatomical complexity of the region. Further clinical experience is warranted to validate and refine this technique.

Endoscopy_UCTN_Code_TTT_1AO_2AG_3AD
